# Classification, location, and intensity of granules in retinal pigment epithelium following sodium iodate injection in rat animal model

**DOI:** 10.22038/IJBMS.2023.71194.15465

**Published:** 2024

**Authors:** Mahboube Mousavi, Aliasghar Mousavi, Behnam Jamei, Hamidreza Sameni, Sam Zarbakhsh, Hamid Aboutaleb Kadkhodaeian

**Affiliations:** 1 Nervous System Stem Cells Research Center, Semnan University of Medical Sciences, Semnan, Iran; 2 Department of Anatomy, Faculty of Medicine, Semnan University of Medical Sciences, Semnan, Iran; 3 Visual Health Center, Semnan University of Medical Sciences, Semnan, Iran; 4 Neurosciences Research Center, Iran University of Medical Sciences, Tehran, Iran

**Keywords:** Age-related macular – degeneration, Fluorescent light intensity, Lipofuscin, Melanin, Sodium iodate

## Abstract

**Objective(s):**

Age-related macular degeneration (AMD) is one of the eye diseases that can affect a person’s central vision. Retinal pigment epithelium (RPE) cells are damaged in this medical condition and some pigments are presented in these cells. Here, we aimed to investigate melanin and lipofuscin granules of RPE cells as a precursor of AMD.

**Materials and Methods:**

Hooded rats (n=18) were divided into two groups and received 100 μl of sodium iodate (SI) into the retro-orbital sinus of their eyes at 40 and 60 mg/kg doses. The total number of melanin and lipofuscin granules, different types of granules, cytoplasmic dispersion of granules as well as morphological changes in the shape and number of nuclei of RPE cells were evaluated over the course of 1-30 days.

**Results:**

The total number of melanin pigments increases over time at a dose of 40 mg/kg and decreases at a dose of 60 mg/kg. Also, the total number of lipofuscin granules in 40 mg/kg increases over time and decreases in 60 mg/kg. Autofluorescent intensity (AF) is also increased at 40 mg/kg, but at 60 mg/kg, the highest intensity is on day 7. Also, the highest number of multinucleated giant cells was on day 7 at 60 mg/kg and the most changes in cell appearance due to sodium iodate injection were seen on the first day after injection.

**Conclusion:**

We demonstrated that granules and autofluorescent intensity appear to decrease at high doses of sodium iodate, which is similar to the advanced stage of the AMD disease, where the number of granules and AF intensity increase in the middle and even early stages of the disease.

## Introduction

AMD is the most important and common cause of blindness in people over 40 years of age in the world (1-3). It is also the most important cause of incurable low vision. The prevalence of this disease is about 8.7% of the worldwide population and it is estimated that by 2040 this rate will reach more than 280 million people (4). AMD is seen in two forms, dry and wet. In the dry type, RPE cells are damaged and photoreceptor cells are destroyed, which impairs a person’s central vision (5).

RPE is a highly polarized epithelial layer located on the back of the eye between the retina and choroid layer. This layer has many important roles such as supporting photoreceptors, visual cycle, phagocytosis, metabolites transportation, light absorption, and secretion of many proteins which are important to retinal health (6, 7). Many studies have reported that sodium iodate can disrupt the function of RPE cells and make an AMD-like animal model, specifically in rodents (8-12). It is known for its toxic effect on PRE cells which leads to RPE cell necrosis, followed by photoreceptor apoptosis. SI is thought to directly affect the RPE cells with secondary effects on photoreceptors and the choriocapillaris and has been shown to induce the production of reactive oxygen species contributing to damage in RPE cells. One of the important effects of SI on RPE cells is the rise in the conversion of glycine to potentially toxic glyoxylate by melanin. 

In general, RPE cells have different types of pigmented and non-pigmented organelles, including lipofuscin, melanolipofuscin, melanosomes, and mitochondria. Among them, lipofuscins and melanolipofuscins have AF (13). Lipofuscin-induced AF can be observed using short wavelengths (488 nm excitation) and melanin can be seen by near-infrared wavelengths (785 nm excitation). Studies have shown that melanin is the first granule at birth in human RPE cells, So the autofluorescent granules appear in the first two years after birth, and very few lipofuscins are found in children younger than 10 years old (14). Lipofuscin is a complex mixture of fluorophores and is detectable by chromatography and mass spectrometers. Only a few bis-retinoid fluorophores have been identified mostly in mice (15). These particles contain the damaged cell membrane of photoreceptors and enter RPE cells inside the lysosome, (16). Lipofuscin is slowly disappearing by RPE cells in that area as the photoreceptor cells die. The remaining bodies, which are converted to lipofuscin, retain their destructive enzymatic activity and are deposited on Bruch’s membrane (17-19). Accumulation of lipofuscin in RPE cells causes emerging fluorescent light spontaneously, and the higher accumulation of liposuction, results in more intensity output. However, with the death of RPE or photoreceptor cells or due to extensive photooxidation / photonic degradation of bis-retinoid compounds after the age of 70, the amount of AF light should diminish or fade. Studies show high AF and an abundant amount of lipofuscin granules in RPE might be a marker of disease or retinal stress (20). However, histological studies of the human eye in healthy individuals and AMD sufferers do not support the hypothesis of lipofuscin-rich RPE cell death (13). Therefore, determining the intensity of light emitted based on tissue information can be effective in determining the extent of damage and the degree of disease and cell destruction (15). The use of techniques such as confocal scanning laser ophthalmoscope (cSLO) (15), Optical coherence topography (OCT), and fundus autofluorescence (FAF), which indirectly provide information on melanin and lipofuscin, in general, provides RPE texture status item. However, the study of cellular changes by these devices cannot be evaluated. Combining AF with Adaptive optics-based techniques now allows direct observation of individual RPE cells. These techniques are being developed to allow more accurate observation of RPE cells. However, they have limitations and cannot provide a good image of eye diseases (21). The tissue basis of FAF and age-related and disease-related autofluorescents has recently been investigated. In destructive diseases such as age-related macular degeneration, RPE cells generally tend to lose AF due to the loss of autofluorescent granules, although due to the accumulation of granules, there are highly hyperfluorescent regions within the cells (13). Therefore, in this study, we aimed to compare the AF intensity of light emitted from RPE cells damaged by sodium iodate in different doses and at different time intervals. 

## Materials and Methods

***Animals***

Hooded rats (n=18) (Razi Institute, Iran, Tehran), weighing 250–300 g, were divided into two groups, comprising 40 mg/kg (n=9) = low dose; 60 mg/kg (n=9) = high dose of sodium iodate. Animals were housed under standard laboratory conditions: 12-hr light/dark cycles at 20 °C. All experimental procedures were performed in accordance with the Association for Research in Vision and Ophthalmology (ARVO) guidelines for the use of animals in ophthalmic and vision research. The study was approved by the Ethics Committee of Semnan University of Medical Science, Semnan, Iran with approval ID IR.SEMUMS.AEC.1401.001.

***Creating an RPE cell degeneration model***

To achieve the RPE injury model, a retro-orbital sinus injection was performed, as previously described (9, 22, 23). In brief, sodium iodate (SI) (Sigma-Aldrich, CAS Number 7681-55-2, Linear Formula: sodium iodate, MW.:197.89, Steinheim, Germany) was diluted in sterile phosphate-buffered saline (PBS) to a final concentration of 5%, filtered, adjusted to pH 7.2 and kept at 4 °C. Then, 40 and 60 mg/kg sodium iodate was injected into the left retro-orbital sinus. Rats were anesthetized with an intraperitoneal injection of ketamine and xylazine (40/ 4 mg/ kg) and following complete anesthesia were positioned on a warm pad (25 °C) and placed in a lateral anatomical position, with the left eye facing up. A 30-gauge, 1-in insulin needle was inserted into the medial canthus, deep into the vessels behind the eye, at a 45^o^ angle to the nose. Sodium iodate (100 μl) was injected gently into the retro-orbital sinus vessels. 

***Tissue preparation and imaging***

One day (n = 3), seven days (n = 3), and 30 days (n = 3) after injection of sodium iodate, eyes were enucleated from three rats randomly chosen from each group. These animals were euthanized under deep anesthesia (ketamine/xylazine at 500/50 mg/kg, IP), during which time the eyes were enucleated and fixed in paraformaldehyde 4% for 2 hr at 4 °C. Corneas, lenses, and neural retinas were removed from each fixed eye. For whole mounts, four radial cuts were made in each eyecup. AF microscopy was performed using an excitation wavelength of 498 nm of RPE sheet with an AF microscope (Olympus IX71 / IX81, Tokyo, Japan). To calculate the fluorescence intensity of RPE cells, the central part of whole mounts was picked, and following their preparation, 488 nm excitation light was irradiated to the samples using a fluorescent microscope with magnification of 40 X and 100 X, and the image of RPE layer was taken. Images were analyzed using the Image J software.

***Specify the granules***

Using FIJI (an open-source image processing package based on ImageJ2), the boundaries of each cell were marked manually by a line, and the area and height (x, y, length, and angle) of the RPE cell were reported. The granules were counted and categorized using a custom FIJI plugin. For a more detailed study of the differences in the cytoplasmic granules’ distribution, the image stack for each layer and cell was performed on the z-axis, and four regions (C1-C4) were identified, where C1/ C2 refers to the apex, C3 the middle, and C4 the basal part of each cell. All detectable granules in the RPE cell were labeled and counted.

***Granule classification***

The classification of granules were performed with some modifications (24). Initial RPE analysis on the z-stack identified different types of granules. Based on the AF pattern of each granule and structural properties (round, oval, or elongated), 5 types of phenotypes were identified. These phenotypes were used in subsequent investigations. For each cell, the total number of granules as well as melanin and lipofuscin granules per cell were reported.

***Determination of total AF intensity of RPE layer***

Determination of AF of the RPE layer was imaged by a fluorescent microscope with a wavelength of 488 nm excitation. Then the images were converted to RGB stack (an additive color model in which red, green, and blue light is added together) with a custom FIJI plugin, and histogram and plot profile analyses were performed. Also, through the volume viewer, the RPE layer and its cells were shown in three dimensions in the x, z, and y axes, and the position of the pigments and their intensities were examined from the top to the base of the RPE cells. RPE fluorescent light intensity was also calculated using FIJI. The calculated light intensity includes the light intensity emitted from all the pixels in all the layers of the apex to the base of the RPE cell, which is shown as an image.

***Data analysis***

The obtained data were analyzed by GraphPad Prism 8.0 software and one-way analysis of variance (RM one-way ANOVA) with *post hoc* Tukey’s was used. The significance level in these analyses was considered *P*<0.05. The Paired Two-tailed t-test was also used to evaluate the data of each light intensity between two different doses of sodium iodate.

## Results

***Total number of cytoplasmic granules of RPE cell***

**Figure 1:**
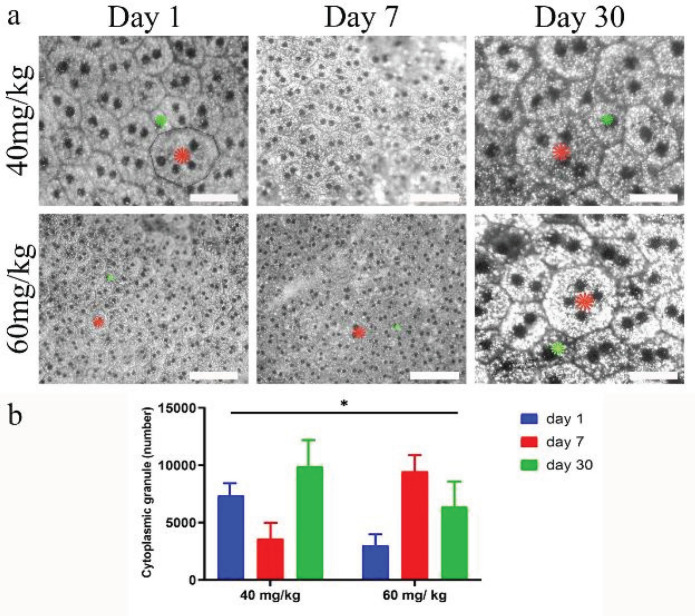
a, The figure shows that there is a difference between rat retinal pigment cells containing high and low granules. Stars indicate cells with large (red) and low (green) granules. A single RPE cell is shown and its plasma membrane is marked with adjacent cells. b, the graph shows that there is a significant difference between groups over the day 1 to day 30 period

To determine the effect of SI on RPE cells, in the first stage, we evaluated RPE cells with high or low granules and the total number of granules, cell surface, and number of granules per cell surface, and cells with high or low granules in the central part (Figure 1). The index of RPE cells with low granule content is a high melanin-to-lipofuscin ratio and low granule ratio. Data showed that in low dose of sodium iodate, about 11,331 granules were calculated, which filled 10–49% of the cell surface while in high dose about 11089 granules were counted, which covered 39–56% of the cell surface. The most remarkable feature in both high and low doses was fluctuation in total granule number. The results illustrated that total granules rose in low dose whereas they collapsed in high dose. With regard to the total number, there was a greater decrease in higher dose than lower dose from day 1 to day 30. It is clear that higher dose of SI dropped the total granules in RPE cells during this time. The number of granules per unit area and the percentage of cell surface are shown in Table 1.

***Classification of cytoplasmic granules***

**Figure 2:**
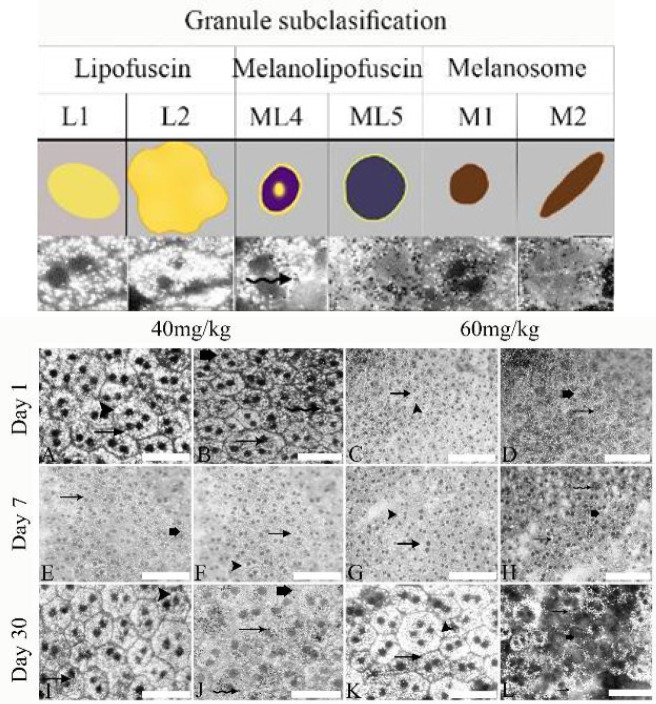
Different types of rat retinal pigment cell granules

The results of the first part raise the question of whether all the granules in the RPE cell are of the same type or are different types. To answer this question, we designed another test in which we categorized the types of cytoplasmic granules (Figure 2). The results showed that there were 5-6 types of cytoplasmic granules based on the shape, size and pattern of AF, which included lipofuscin (in two types: L1 monolithic granules and L2 irregular and larger aggregates with AF), melanolipofuscin [In two types: large round granules (ML1 and bull’s-eye-shaped granules (ML2)) and melanosomes (in two rounds, M1 and spindle-shaped M2)]. Next, the effect of sodium iodate on the number and variety of granules in RPE cells was investigated. The most outstanding feature at both doses between day one and day 30 after injection was that all granules were present in RPE cells, meanwhile there was a statistically significant difference in the total number of melanin and lipofuscin in both doses.

***Total number of melanin and lipofuscin granules***

**Figure 3:**
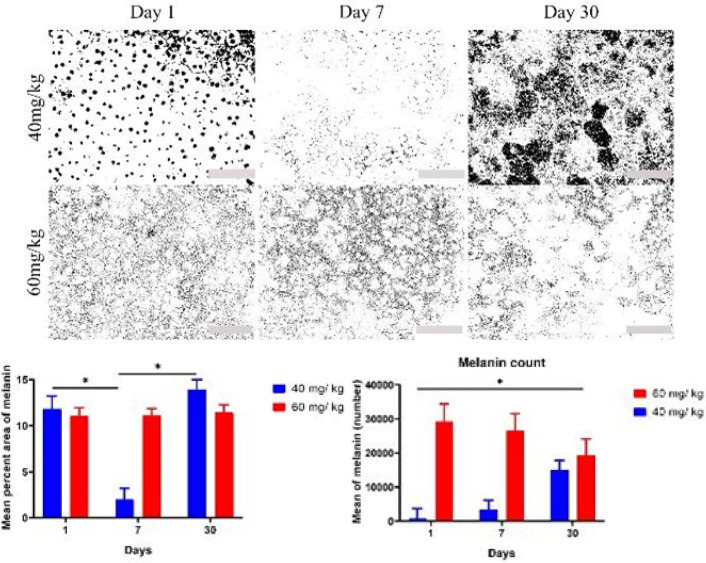
Figures reveal changes in rat melanin count between two different doses over the day 1 to day 30 period after SI injection

**Figure 4:**
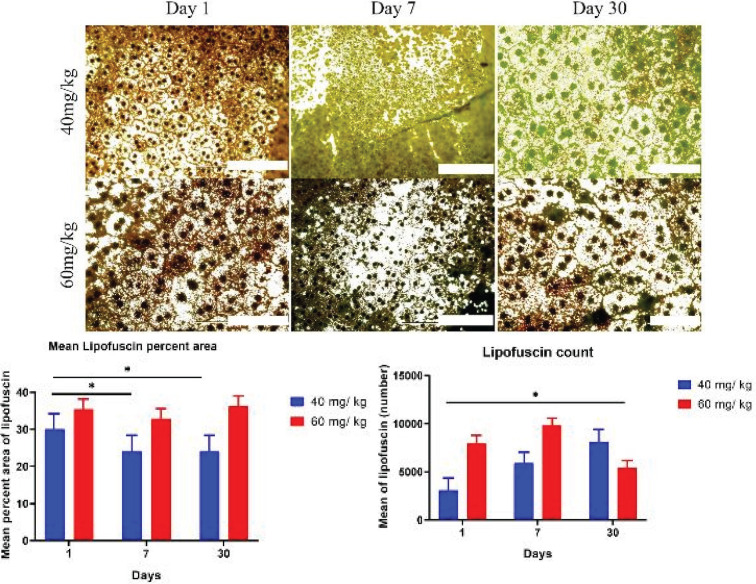
Images illustrate the changes in rat lipofuscin granules between two different doses from day 1 to day 30 after injection of sodium iodate

In the next step, to determine more accurately the effect of SI on increasing or decreasing the amount of RPE cell granules between day one to day 30, we designed another test in which the total number of melanin and lipofuscin granules and the ratio of melanin + melanosome to lipofuscin were calculated. Figures 3 and 4, and Tables 2 and 3 show the results of this test. The results showed that there was a statistically significant increase in the number of melanin granules at low dose from day 1 to day 30 after injection, meanwhile, at high dose there was a gradual decline in the number of melanin granules. It is estimated that the deterioration of melanin in high dose of SI was more than in low dose. 

The results of lipofuscin showed that there is a statistically significant increase in the number of lipofuscin granules at low dose from day 1 to day 30. However, at high dose, the highest point of lipofuscin was on day 7. The main remarkable point was that total lipofuscin granules in the low dose at 30 days was more than in the high dose. It was evident that the surge in lipofuscin in the low dose at the end of day 30 was more than in the high dose. All in all, the number of both melanin and lipofuscin granules increased in the low dose in comparison with the high dose. 

***AF intensity of lipofuscin***

**Figure 5:**
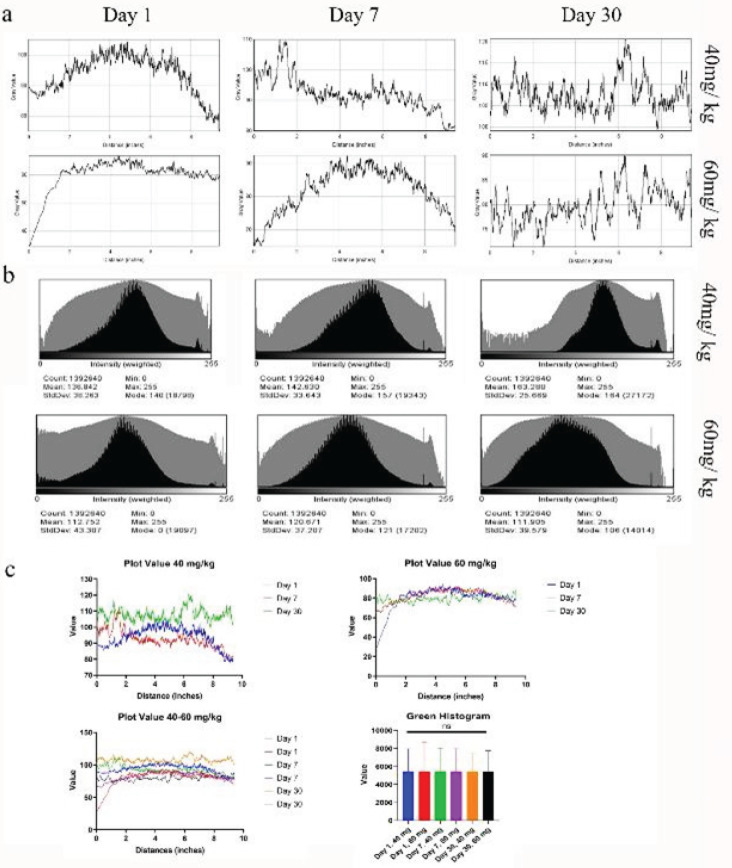
Lipofuscin hyperfluorescence in rat RPE cells at low and high doses of SI

The results of lipofuscin led us to evaluate the AF intensity of RPE cells between day 1 and day 30 after SI injection. Figure 5 and Table 4 show the results of this evaluation. The results showed that there was a statistically significant difference in the full value of AF intensity of RPE cells at low dose of SI (Table 4 and Figure 5) while at high dose it was fitful. Moreover, the green histogram and plot value revealed a slight elevation in AF at low dose. The main noticeable feature was that AF intensity at high dose was not significant in contrast to low dose between days 7 and 1. It is predicted that it will be due to the death of RPE cells at that time point. Overall, although AF of RPE cells collapsed in both doses over the period of time, there was no statistically significant difference between low and high doses of SI. 

***Cytoplasmic dispersion of granules***

**Figure 6:**
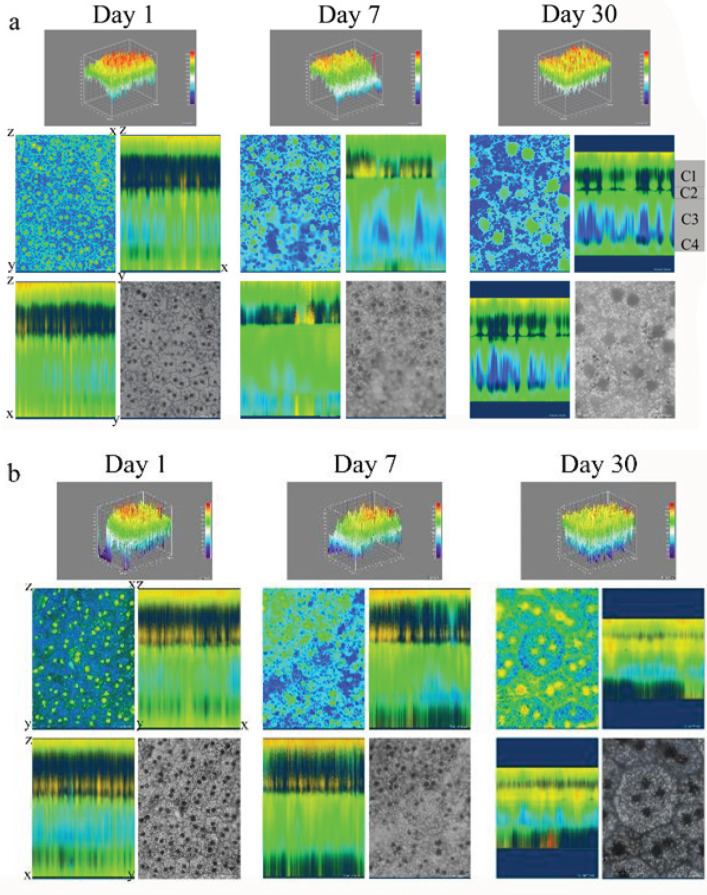
Dispersion of granules in the cytoplasm of rat RPE cells

The results of Figure 5 raise the question of how melanin and lipofuscin pigment dispersed in the cytoplasm of RPE cells following SI injury. To answer this question, we designed another test in which we examined the distribution of melanin and lipofuscin in the cytoplasm of RPE cells. Figure 6 shows the results of this test. The results of three-dimensional volumetric examination of the RPE layer (3D volume viewer) and three-dimensional intervention (3D interactive) showed that the granules are located in four parts in the cytoplasm of the RPE cell. 1. Melanin was concentrated in the microvilli and apical part of the cytoplasm (regions C1 and C2), 2. Lipofuscin was the middle part (region C3), and 3. Predicted mitochondria in the basal part (region C4). It was clear that at low dose of SI and on day 1 (Figure 6 top panel), the amount of melanin was very high in comparison with day 7 and located as a compact black row in the apical side of the RPE cells. However, the amount of lipofuscin was very low in contrast to melanin and located mainly in the C3 region. Moreover, the amount of lipofuscin increased on days 7 and 30 as opposed to day 1. Likewise, at high dose of SI, there was a greater decrease in melanin on day 30 in contrast to days 1 and 7. In addition, the lipofuscin declined on day 30 compared with days 1 and 7. The main remarkable feature was that SI did not interfere with the distribution and arrangement of cytoplasmic granules in RPE cells.

***Number of nuclei***

**Figure 7:**
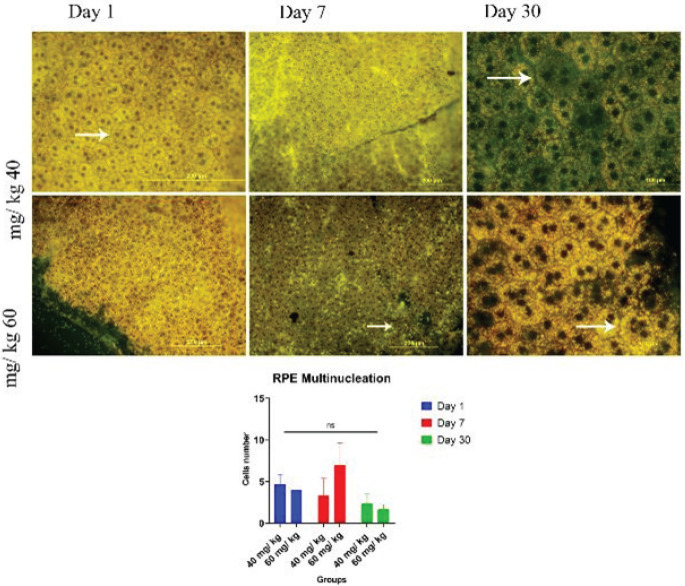
Difference in number of rat RPE cell nuclei after SI injection into retro-orbital sinus

Generally, RPE cells are divided into two types, 1- single-nuclei and 2- bi-nuclei. The former is seen usually at the periphery and the last one in the central part of the RPE layer. The number of multinucleated cells in the central part increases with age rather than in the peripheral part. We designed a study to investigate the effect of SI on increasing the number of multinucleated cells in the central part of the RPE flat mount. The results (Figure 7) showed that the number of giant multinucleated cells at low dose of SI decreased during the first day to day 30 after injury. However, at low dose, there was a greater increase in the number of multinucleated giant cells on day 7 than on day 30. It was clear that the giant cells were seen in both doses in the shape of three or four nuclei. It is predicted that seven days after injection of high dose of SI is similar to physiological aging in terms of multinucleated cells.

***Cell morphology***

**Figure 8:**
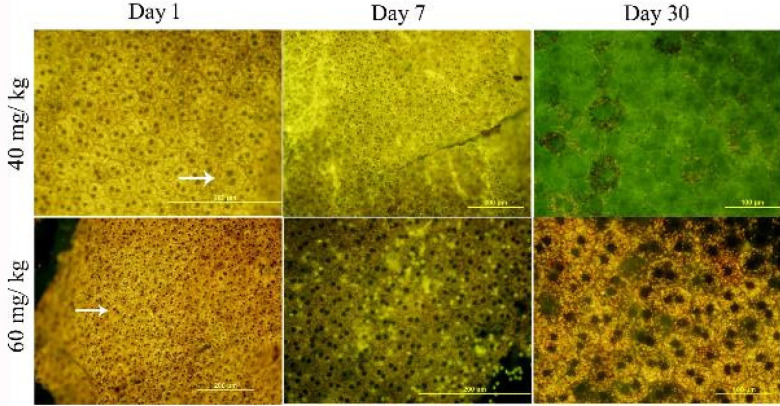
The shape of rat RPE cells changes after injury at two SI doses. White arrows show RPE cells with different morphology

The results present information about the cell shape changes following injury by SI over the day 1 to day 30 period (Figure 8). The main noticeable point was the large RPE cells with cytoplasm and swollen nuclei and more unusual polygonal shapes (octagonal or nine-sided) at day 1 in both high and low doses of SI compared with days 7 and 30. It was clear that the damage-related cell shape changes had been on the first day after the SI injection.

## Discussion

In this study, we investigated cytoplasmic granules and AF intensity emitted from the RPE layer after injection of SI in two different doses. The total number of cytoplasmic granules, different types of granules, the total number of melanin and lipofuscin, lipofuscin AF intensity, the cytoplasmic distribution of granules, as well as the morphological changes in the shape and number of RPE cell nuclei, were investigated. The results showed that at a low dose, the total number of granules increases over time, and at the high dose, the highest amount of granules is seen on day 7 after SI injection. The total number of melanin also increases over time at low dose and decreases at high dose. Moreover, the total number of lipofuscin in low dose increases over time while decreasing in high dose. Besides these findings, 5 types of granules were identified inside the cytoplasm of SI-damaged RPE cells in the apical, middle, and basal parts of RPE cells. We also showed that the intensity of AF is increased at the low dose, however, the highest intensity at the high dose is on day 7. Furthermore, the highest number of multinucleated giant cells was on day 7 at high dose, also the most changes in cell morphology due to SI damage were on the first day.

The RPE layer plays an important role in the health of the retina. Changes in the RPE layer are seen in many degenerative retinal diseases such as AMD. One of the factors contributing to that disease is changes in RPE cytoplasmic granules. These pigments begin to form in the embryo and increase in number in the cytoplasm after birth due to several factors, including cell damage and aging, and one of the main diagnostic signs of AMD is the amount and changes of cytoplasmic granules (25). In the present work, three types of pigments were observed in the cytoplasm of cells in the central part of RPE, and this is in line with a study that shows the presence of three types of melanin, lipofuscin, and melanolipofuscin in these cells (24, 26). Moreover, researchers (24) identified 9 phenotypes for human RPE cell cytoplasmic granules in individuals between the ages of 16 and 90 years. RPE granules exhibited two phenotypes for lipofuscin, two phenotypes for melanin, and 5 phenotypes for melanolipofuscin. In our study, two types of melanin and lipofuscin phenotypes and two phenotypes of 5 melanolipofuscin phenotypes were found in the central RPE cells. However, it seems that other melanolipofuscin phenotypes may be detectable if a more appropriate microscope is used. In this study, except for two melanolipofuscin phenotypes, another phenotype was identified that is not categorized in the initial classification. This is in line with the findings of Bermond *et al*. (24) that showed this form of lipofuscin is in fact the intermediate state between pure melanosome and pure lipofuscin and is seen during the evolution of lipofuscin and conversion of melanosome to lipofuscin. Studies have shown that at birth in humans, RPE cells lack lipofuscin and melanolipofuscin and have only melanosome, and autofluorescent granules appear in the first two years after birth, and very few lipofuscins are found in children younger than 10 years old, which is probably the reason for the gradual development of fovea during the first year of birth, increase in the density of photoreceptors during the first ten years of birth, and the accumulation of non-absorbable materials and autofluorescent lipofuscin granules (14). Therefore, decreased melanin and increased lipofuscin are the main symptoms of aging in RPE cells. Similarly, in our study, the results of total granules, melanin, and lipofuscin reveal that, 30 days after injection of the low dose of SI, the total number of granules, melanin, and lipofuscin increases over time, while decreasing in high dose. Although lipofuscin declines in high dose, it is predicted that these cells probably undergo a degenerative process. The main remarkable point is that the RPE cells in high dose die after day 30 (9). Accordingly, the transformation of melanin to melanolipofuscin at the high dose of SI is the main cause of melanin drop at the high dose and lipofuscin rise at the low dose. Consistent with the results of our research, other studies have reported an increase in lipofuscin and a decrease in melanin granules in all areas of the retina (fovea, around the fovea, and near the periphery) (16). However, other researchers have reported only a reduction in fovea melanin rather than an augmentation in lipofuscin (27), or an increase in lipofuscin from the fovea to the periphery, with the highest levels of lipofuscin in human specimens in the prefoveal and superior-temporal parts (15, 28). 

Regarding the cytoplasmic distribution of pigments in this study, we showed that melanin and melanolipofuscin are located in the apex of the cell, lipofuscin in the middle, and mitochondria in the basal part of the RPE cell. In this regard, Bermond *et al*. (24) showed that melanolipofuscin is located in the apex to the middle part, and lipofuscin in the basal-lateral and basal parts. There is also a small amount of melanin in the apex and the fourth phenotype of melanolipofuscin in the apex. Also, injection of SI in different doses slightly disrupts the cytoplasmic arrangement of pigments. In this regard, it was shown that although the number of lipofuscin pigments increases with age, their distribution and dispersion do not change with age (24).

Lipofuscin and melanolipofuscin pigments have AF due to excitation by short wavelengths, and melanin pigments also have AF as a result of excitation with infrared wavelengths (15, 24). Remarkably, in RPE cells, riboflavin-bound melanin does not emit AF, but when melanosome reacts with lipofuscin, they oxidize and emit fluorescence (infrared wavelength), which is seen in the Stargardt disease (14). Studies have shown that fundus AF increases linearly with age. Although light intensity varies, it stops after age 70 and in diseases such as geographic atrophy due to loss of photoreceptors and RPE, or change in the emitted AF due to photooxidation/photodegradation of bis-retinoid compounds (15). However, we showed that at the high dose, the maximum amount of AF is on day 7 and decreases gradually from day 7 to day 30, which is predicted to be RPE cell death. However, at the low dose, AF has an upward trend, which indicates the transformation of melanin to melanolipofuscin and lipofuscin (a symptom of RPE cell aging). Although studies have shown that lipofuscin usually forms in photoreceptors and is transported to RPE cells (15), the production of lipofuscin and melanolipofuscin from melanin has also been recently reported (24). In other words, some phagosomes containing bis-retinoids that are not digestible, combine with melanosomes to form melanolipofuscins. It has been demonstrated that if the amount of zinc (Zn) is reduced in the diet, melanin in melanosomes that combine with phagosomes containing digestible bisretinoids can produce lipofuscin and melanolipofuscin (14). Therefore, in abnormal RPE cells where melanin decreased and lipofuscin increased (as in the present study), short-wavelength AF increased and near-infrared AF decreased. This finding is in line with studies showing that short wavelengths (488 nm excitation) are emitted from lipofuscin pigment, and infrared wavelengths (785 nm excitation) are emitted from melanin pigment (29). Examination of melanin in RPE cells (30) shows that this pigment is associated with eye diseases such as AMD and RP (29). For example, hyperfluorescence rings are seen in the retina of RP patients. These rings are a useful marker for assessing RPE, and as the disease progresses, the rings also develop. Although short wavelengths are very important in the diagnosis of this disease, recently the use of infrared wavelength has been proposed as a diagnostic tool. Studies show that there is a very strong correlation between the entropy of hyperfluorescence rings and the light intensity of infrared waves, while there is a weak negative correlation between entropy and short wavelength waves (29). Therefore, the use of infrared waves (to detect melanin) along with short wavelengths (to detect lipofuscin) can be very effective in monitoring RP patients. 

In this study, 5 types of cytoplasmic granules were identified in RPE cells damaged by SI. This is in line with others that showed that there are different types of granules in the cytoplasm of RPE cells including lipofuscin, melanin, melanolipofuscin, and mitochondria. Lipofuscin and melanolipofuscin pigments emit AF after stimulation by blue light, and AF of RPE cells depends on factors such as proximity to fovea and age (13). 

RPE cells in the periphery are very diverse in shape and size. Some of these cells are long and irregular, and some cells are polygonal (pentagonal or hexagonal). The cells of the central part are more uniform and are usually pentagonal or hexagonal. In general, the cells in the peripheral part are larger than the central, and their size usually increases with age in both parts. In AMD, RPE cells are destroyed and the remaining cells try to fill the space of the damaged cells. In the present study, we showed that the number of multinucleated giant cells increased in high dose seven days after sodium iodate injection. These results are similar to those of Chen *et al*. in 2016, which showed that as C57BL / 6J mice grew up to 24 months of age, the number of RPE cells decreased and the remaining cells became larger, multinucleated to maintain layer uniformity, and overall size of the cells increased. These researchers showed that multinucleated RPE cells are formed due to defects in cytokines following exposure to the outer part of the oxidized photoreceptors (31). In our work, we demonstrated that the morphology of RPE cells changes on the first day after sodium iodate injection, and the cytoplasm and cell nucleus become swollen. Other research shows that the presence of swollen and giant multinucleated cells is a reason for the process of cell repair after injury. They state two pathways for RPE cell repair after injury: 1- When a single cell is damaged, in which case the cells adjacent to the damaged cell become enlarged and swollen and move toward the damaged cell and repair it. 2- When several RPE cells are damaged. In this condition, the surrounding cells become large, swollen, and multinucleated and repair the damaged cells. So, giant multinucleated cells are formed or remain single-nucleated and die (patchy loss) under the influence of cellular stress.

**Table 1:** The total number of melanin, total area, average size, percentage area, and mean number of melanin in the RPE cells during the time course and different doses of SI

Count		Total area		Average size		% Area		Mean	
40 mg	60 mg	40 mg	60 mg	40 mg	60 mg	40 mg	60 mg	40 mg	60 mg
Day one									
**11331**	3004	28.355	38.165	0.003	0.013	42.219	56.827	84.676	106.438
**4837**	3004	27.377	38.165	0.006	0.013	40.764	56.827	84.933	106.438
**5908**	3004	29.601	38.165	0.005	0.013	44.075	56.827	120.377	106.438
Day seven									
**2189**	8931	32.981	26.479	0.015	0.003	49.108	39.426	121.344	110.108
**5027**	11089	30.979	29.17	0.006	0.003	46.126	43.433	77.306	115.597
Day thirty									
**5997**	4429	28.524	24.807	0.005	0.006	42.472	36.937	121.548	100.879
**11263**	3966	7.327	26.011	6.51E-04	0.007	10.91	38.73	76.281	106.617
**10501**	5423	9.992	24.652	9.52E-04	0.005	14.877	36.706	73.43	95.468
**10975**	11705	12	22.066	0.001	0.002	17.868	32.855	78.105	112.3
**10809**	20.033	12.763	20.033	0.001	0.002	19.003	29.829	81.636	61.767

RPE: Retinal pigment epithelium

Error bands, SD for 3 rats per each time course and each dose (* *P*<0.05).

Scale bar: 100 μm and 200 μm

RPE: Retinal pigment epithelium

The top panel shows a schematic diagram of the types of cytoplasmic granules and their samples in RPE cells

The bottom panel shows the types of granules in two different doses of SI on days one, seven, and thirty. A, C, E, G, I, and K represent two types of lipofuscin pigment and B, D, F, H, J, and L represent the pigment of melanosome and melanolipofuscin.

Scale bar: 100 μm and 200 μm

L: Lipofuscin; ML: Melanolipofuscin, M: Melanosome; RPE: Retinal pigment epithelium; SI: Sodium iodate

The top panel shows the changes in melanin and the bottom panel shows the total number of melanin and the percentage of cell surface occupied by melanin. At low dose, the amount of melanin increases over time, but at high dose, the trend decreases. Error bands, SD for 3 rats per each time course and each dose (* *P*<0.05).

Scale bar: 100 μm and 200 μm.

SI: Sodium iodate

The top panel shows changes in lipofuscin and the bottom panel shows the total number of lipofuscin and the percentage of cell surface occupied by lipofuscin. Error bands, SD for 3 rats per each time course and each dose (* *P*<0.05).

Scale bar: 100 μm and 200 μm.

**Table 2:** Mean of melanin granules, cell surface, percentage of cell surface, and mean during the time course and different doses of SI

Count		Total area		Average size		% Area		Mean	
40 mg	60 mg	40 mg	60 mg	40 mg	60 mg	40 mg	60 mg	40 mg	60 mg
Day one									
**829**	29149.67	7.933	7.457667	0.01	2.55E-04	11.811	11.10433	44.307	81.20167
Day seven									
**3435**	26562.25	1.359	7.48525	3.96E-04	0.000285	2.023	11.14525	95.155	68.15475
Day thirty									
**15042.2**	19434.6	9.3396	7.6842	5.72E-04	3.93E-04	13.9064	11.4418	44.843	83.6406

**Table 3:** Mean lipofuscin granules, cell surface, cell surface percentage and mean by day and dose

Count		Total area		Average size		% Area		Mean	
40 mg	60 mg	40 mg	60 mg	40 mg	60 mg	40 mg	60 mg	40 mg	60 mg
Day one									
**3049**	7958.333	20.183	23.78567	0.007	0.003333	30.052	35.41667	134.884	102.352
Day seven									
**5914**	9871.75	16.148	22.0595	0.003	0.00225	24.044	32.846	125.989	83.65375
Day thirty									
**8105.667**	5415.2	16.16333	24.3394	3.32E-03	0.0046	24.06683	36.2406	88.31883	104.8972

a, b show plot value and green histogram of AF intensity of RPE cells. c shows how AF intensities differed in low and high doses of SI. Error bands, SD for 5 rats per each time course and each dose (* *P*<0.05, ** *P*<0.01, *** *P*<0.001, **** *P*<0.0001).

RPE: Retinal pigment epithelium; SI: Sodium iodate; AF: Autofluorescent intensity

**Table 4:** Statistical analysis of plot value of AF intensity of RPE cells using a two-tailed t-test

Days	40 mg/kg		60 mg/kg	
*P*-value summary	Significant	*P*-Value	Significant	*P*-Value
Day 7 vs Day 1	****	<0.0001	****	<0.0001
Day 30 vs Day 1	****	<0.0001	ns	0.6561
Day 30 vs Day 7	****	<0.0001	****	<0.0001

AF: Autofluorescent intensity; RPE: Retinal pigment epithelium

a indicates low dose and b shows high dose of SI. The first row (in each panel) shows how granules are arranged in volumetric three-dimensional, and the middle and lower rows in a and b show the intervening three-dimensional arrangement of granules in the cytoplasm relative to the xz, yz, and xz axes, respectively. Blue color represents lipofuscin, black indicates melanin, and blue-black shows mitochondria and cell nuclei.

RPE: Retinal pigment epithelium; SI: Sodium iodate

The arrows represent the giant multinucleated RPE cells. Error bands, SD for 3 rats per each time course and each dose (* *P*<0.05, ** *P*<0.01, ***P<0.001, **** *P*<0.0001).

Scale bar: 100 μm and 200 μm

RPE: Retinal pigment epithelium; SI: Sodium iodate

Scale bar: 100 μm and 200 μm

RPE: Retinal pigment epithelium; SI: Sodium iodate

## Conclusion

The use of AF intensity emitted from the cytoplasmic pigments of RPE cells can be used to determine the severity of damage to these cells. At a high dose of sodium iodide, which is similar to the advanced stage of AMD disease, granules and AF intensity appear to decrease, and in the middle and even early stages of the disease, the number of granules and AF intensity increase. The data from this study can be used to diagnose the stages of AMD disease.

## Authors’ Contributions

M M contributed to material preparation, data collection, and writing the primary draft. A M, B J, H S, and S Z prepared the study material. HA K conceived the study and design and contributed to material preparation, data collection, analysis, and writing the manuscript. All authors read and approved the final article.

## Funding

This work was supported by Grant Research from Semnan University of Medical Sciences, Iran (number IR.SEMUMS.AEC.1401.001).

## Declaration of Interest

None.

## Data Availability

The datasets generated and/or analyzed during the current study are available in this article.

## Ethical Approval

Eye samples were collected and carried out according to the Ethical Standards of the Responsible Committee on Animal Experimentation (institutional and national) and the Helsinki Declaration of 1975, as revised in 2008.

## Conflicts of Interest

The authors have no conflicts of interest.
